# Evaluation of Reirradiation in Locally Advanced Head and Neck Cancers: Toxicity and Early Clinical Outcomes

**DOI:** 10.1155/2018/8183694

**Published:** 2018-03-26

**Authors:** Amit Bahl, Arun Singh Oinam, Arun Elangovan, Satinder Kaur, Gaurav Trivedi, Roshan Verma, Sudhir Bhandari, Sushmita Ghoshal, Naresh Kumar Panda

**Affiliations:** ^1^Department of Radiation Oncology, Postgraduate Institute of Medical Education and Research, Chandigarh 160012, India; ^2^Department of Otolaryngology, Postgraduate Institute of Medical Education and Research, Chandigarh 160012, India; ^3^Department of Oral Health Sciences, Postgraduate Institute of Medical Education and Research, Chandigarh 160012, India

## Abstract

**Objectives:**

Locoregional recurrence is the predominant pattern of treatment failure in advanced head and neck cancers. Reirradiation is a useful modality to treat inoperable head and neck cancer patients with recurrent disease. The aim of the present study was to analyze the treatment toxicity and early clinical outcomes in patients undergoing reirradiation.

**Methods:**

Twenty patients of head and neck cancers with recurrences or second cancers were evaluated. Reirradiation was done using simultaneous integrated boost volumetric modulated arc therapy (SIB VMAT), intensity modulated radiotherapy (IMRT), or conventional radiotherapy using 6MV photons. Dose prescription ranged from 30 to 60 Gy in conventional fractionation.

**Results:**

Seventeen males and three females were evaluated in this analysis. The median age of patients under study was 56.5 years. At time of analysis 8 patients (40%) had a complete response, 7 patients (35%) had progressive disease, and 25% had partial response or stable disease. Grade III-IV mucositis, dermatitis, xerostomia, dysphagia, and trismus were seen in 20%, 20%, 50%, 35%, and 45% patients, respectively, during retreatment. Patients receiving a radiotherapy dose less than 45 Gy showed a higher incidence of progressive disease (*p* = 0.01). The median disease-free survival for patients receiving reirradiation dose of ≥46 Gy was 19 ± 3.3 months (median ± S Error) compared to 8 ± 2.61 months for those with a dose prescription less than 45 Gy (*p* = 0.03). At 18-month follow-up 26% of patients undergoing reirradiation were disease-free.

**Conclusions:**

Our results show improved tumor control using a prescription of doses ≥46 Gy in retreatment setting.

## 1. Introduction

Locoregional recurrence is the predominant pattern of treatment failure in locally advanced head and neck cancers. For nonnasopharyngeal squamous cell cancer of the head and neck 5-year survival rates below 50% are reported [1]. Even in the electively irradiated neck a recurrence rate of 4–11% has been reported in literature [2]. Management of recurrent head and neck cancers involves surgery, chemotherapy, reirradiation, and use of targeted agents like cetuximab either alone or as combination therapy [3, 4]. Brachytherapy and external beam radiotherapy both have been used for reirradiation of surgically inoperable cases. Mucosal toxicity, osteoradionecrosis, nerve injury, and carotid vascular damage associated with reirradiation have traditionally restricted the radiation doses used in such practice using conventional external beam radiation techniques [5, 6]. Intensity modulated radiotherapy (IMRT) and its further refinements like image guided radiotherapy (IGRT) and arc treatment have boosted the practice of reirradiation using external beam radiotherapy because of the highly conformal dose to tumor bearing area and the ability to restrict doses received by surrounding organs at risk [7, 8]. Volumetric modulated arc radiotherapy is a relatively new technique and gives comparable dosimetry to IMRT with significantly less treatment time [9]. Chen et al. reported in field control rates of 72% at one year using image guided IMRT for retreatment [7]. In this analysis we report the acute toxicity and early clinical outcomes of reirradiation in our cohort of head and neck cancer patients.

## 2. Material and Methods

Twenty patients of head and neck cancers undergoing reirradiation were included in this retrospective analysis. All the patients had received their first course of radical radiotherapy at our centre using conventional techniques on a Cobalt 60 unit or 6MV linear accelerator and the previous treatment records were available for review. All patients had been evaluated in our multidisciplinary head and neck tumor clinic for surgical resection and found to be inoperable and had been referred for reirradiation.

For radiation treatment planning immobilization was done using perforated thermoplastic casts. Contrast enhanced planning CT scan images were acquired using 3 mm slice thickness and treatment planning was done using Eclipse treatment planning system v11 (Varian Medical system, Palo Alto, CA, USA). The gross tumor volume (GTV) was defined as all gross disease seen on the planning scans. A clinical target volume (CTV) was generated using a 5 mm margin around the GTV. The CTV was expanded symmetrically by 5 mm to create the planning treatment volume (PTV). Elective nodal irradiation was not performed. Treatment plans were generated using 6MV photons with 2–4 arcs using volumetric modulated arc therapy, seven field IMRT, or conventional radiotherapy. Dose prescription ranged from 30 to 60 Gy in conventional fractionation. The spinal cord, brainstem, optic chiasm and nerves, eyes, temporal lobes, carotid vessels, and mandible were contoured as high priority avoidance structures. Since the first course of radiotherapy had been delivered using conventional 2D radiotherapy techniques, the organs at risk were assumed to have received maximal permissible dose. Constraints to critical organs were tailored for each individual patient with an aim to reduce the dose to as low as achievable. Acute toxicity was evaluated using common terminology criteria for adverse events (CTCAE v3) [10] and was defined as occurring within ninety days of treatment. Response evaluation was done using response evaluation criteria in solid tumors (RECIST) [11]. For statistical analysis the data was entered into SPSSv20. Descriptive statistics of all parameters under study were generated. Progression was considered as locoregional increase in disease or distant metastasis. Disease-free survival was evaluated using Kaplan-Meier analysis. Univariate analysis was done to evaluate relationship between variables under study. A *p* value less than 0.05 was considered statistically significant.

## 3. Results

**Figure 1:**
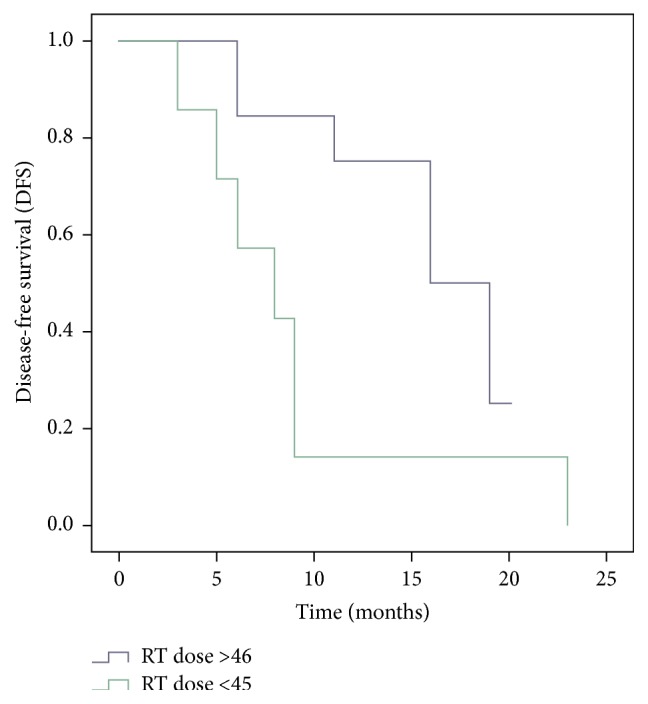
Disease-free survival versus radiotherapy dose.

**Figure 2:**
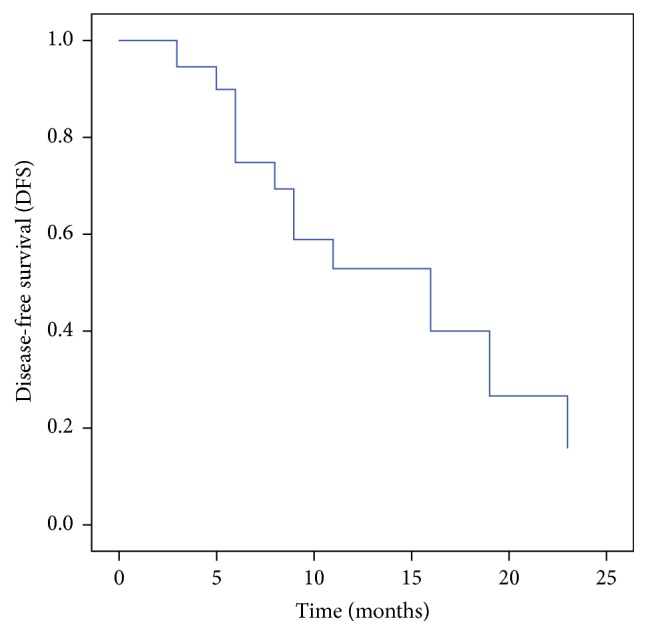
Disease-free survival for the study cohort.

Seventeen males and three female patients were evaluated in this analysis. The median age of patients under study was 56.5 years (range 40–70 years). The treatment characteristics for primary treatment and reirradiation are outlined in Tables 1 and 2, respectively. Second malignancy was diagnosed in 50% cases and the rest were treated for recurrent disease. The median gap between retreatment and the initial radiation was 65 months (range 16–309 months). At time of analysis 8 patients (40%) had a complete response, 7 patients (35%) had progressive disease, and 25% had partial response or stable disease. 65% of patients received a reirradiation dose of ≥46 Gy. A treatment dose of ≥50 Gy was prescribed in 35% of the treated patients. Grade III-IV mucositis, dermatitis, xerostomia, dysphagia, and trismus were seen in 20%, 20%, 50%, 35%, and 45% patients, respectively, during retreatment (Table 3). Trismus (grades 3-4) was the main late toxicity seen in 45% of patients. No osteoradionecrosis or vascular complications were seen till the time of this analysis. Patients receiving a radiotherapy dose of less than 45 Gy showed a higher incidence of progressive disease (*p* = 0.01). The disease-free survival is shown in Figures 1 and 2. The median disease-free survival for patients receiving reirradiation dose of ≥46 Gy was 19 ± 3.3 months (median ± S Error) compared to 8 ± 2.61 months for those with a dose prescription less than 45 Gy (*p* = 0.03). The median survival for the entire cohort was 16 ± 5.2 months. At 18-month follow-up 26% of patients undergoing reirradiation were disease-free.

## 4. Discussion

Surgery is the standard treatment modality for radiation failure cases and recurrent head and neck cancers which are operable [12]. Recurrent disease which is inoperable and not amenable to surgical excision is generally more difficult to manage and is associated with a poor treatment outcome. Treatment modalities in such situations are limited to reirradiation using external beam radiotherapy or brachytherapy, chemotherapy, or targeted agents like cetuximab. Stereotactic radiotherapy has also been evaluated for such treatments with the aim of reducing the treatment volume [13]. Chemotherapy regimens are usually associated with partial responses in up to 30–35% of patients [14]. Vermorken et al. evaluated the role of monoclonal antibody cetuximab and cisplatin chemotherapy in head and neck cancers and reported improved survival from 7.5 to 10.1 months [15].

Reirradiation is based on the premise that normal and critical structures recover some of their tolerance with passage of time [16]. Reirradiation can be however challenging if the earlier treatment has been done with conventional radiotherapy and critical structures have received their full tolerance dose. Janssen et al. recommended reirradiation with curative intent using a dose prescription of at least 46 Gy [17]. Datta et al. reported a better response to reirradiation with a total prescription dose more than 40 Gy [18]. Other authors have used a higher prescription dose of more than 50 Gy and doses up to 60 Gy or higher have been used in retreating regions which are away from earlier high dose prescription zone [19, 20]. In our study 65% of patients received a dose more than 46 Gy with 35% receiving a dose more than 50 Gy with a better outcome for a prescription of at least 46 Gy.

Factors influencing decision making in curative reirradiation include time since previous treatment, earlier radiation dose and technique, location, and volume to be irradiated [21, 22]. PET CT scan is recommended to evaluate the volume requiring retreatment [23]. A time interval of more than 6 months from previous radiation is accepted by some as adequate for retreatment [24], but there is experimental data to suggest that a period of at least 2 years is required for cervical cord to recover from previous radiation dose [16]. The minimum time interval between the two courses of radiation in our cohort of patients was 16 months with a median gap of 65 months. A surface area and volume of reirradiation less than 125 cm^2^ and 650 cm^3^, respectively, have been shown to be associated with better treatment outcome [25]. A margin of 0.5 cm around the gross recurrent disease has been used to generate the reirradiation volume [26]. Biologically effective dose (BED) is another parameter which can be used to evaluate dose to critical organs during reirradiation. For spinal cord the cumulative BED is estimated to be 130–150 Gy [27]. Riaz et al. developed a nomogram based on stage, site of disease, previous surgery, and radiotherapy to predict a response to retreatment and help decision making [22]. Dawson et al. reported 2-year actuarial survival of 32% with retreatment. Severe treatment associated complications were seen in 18% of patients [28]. Langendijk et al. reported a 3-year locoregional control of 22% at 2 years in using dose prescription up to 60 Gy [29]. Chen at al. reported results of using image guidance in IMRT for reirradiation with 2-year rates of control of 65%. Grade 3 or more skin desquamation, dysphagia, and mucositis were reported by them in 57%, 42%, and 23% patients, respectively [7]. IMRT techniques [8] for reirradiation have shown higher local control rates compared to non-IMRT techniques (52% versus 20%). Failures of reirradiation are mainly within the treatment portals and are likely due to the fact that more resistant tumor clonogens are present at the site of recurrence [26].

The present analysis has a small number of patients with a limited follow-up but it reaffirms the use of a reirradiation dose of more than 46 Gy in conventional fractionation for curative retreatment of head and neck cancer patients. The late toxicity profile continues to evolve in patients surviving longer and needs to be further evaluated in our cohort of patients.

Reirradiation is a viable treatment option for inoperable recurrent tumors but a cautious patient selection with judicious treatment planning is required to achieve clinically useful results.

## 5. Conclusions

Our results show improved tumor control using a prescription of doses ≥ 46 Gy in retreatment setting. Manageable acute toxicity was seen with trismus being the most common late toxicity in this analysis.

## Figures and Tables

**Table 1:** Treatment characteristics for the first course of radiotherapy.

Patient characteristic	*n*	Percent
Gender		
Men	17	85%
Women	3	15%
Primary diagnosis		
Anterior tongue	3	15%
Base of tongue	3	15%
Retromolar trigone	2	10%
Maxilla	3	15%
Nasal cavity	3	15%
Larynx	3	15%
Hypopharynx	2	10%
CUP	1	5%
Stage		
I	4	20%
II	8	40%
III	7	35%
IV	1	5%
Treatment		
RT alone	18	90%
CTRT	2	10%
RT dose (mean ± SD Gy)	59.75 ± 5.77	
	(Range 45–66 Gy)	
BED_3_ (mean ± SD Gy)	99.43 ± 10.04	

CUP, carcinoma with unknown primary; CTRT, chemoradiotherapy; BED3, biologically effective dose for *α*/*β* value 3; SD, standard deviation.

**Table 2:** Treatment characteristics for reirradiation.

Patient characteristic	*n*	Percent
Recurrent/new disease site		
Anterior tongue	2	10%
Base of tongue	4	20%
Buccal Mucosa	2	10%
Retromolar trigone	1	5%
Maxilla	4	20%
Floor of mouth	1	5%
Nasal cavity	2	10%
Larynx	2	10%
Hypopharynx	1	5%
Alvelous	1	5%
Stage		
II	5	25%
III	9	45%
IV	6	30%
Radiotherapy technique		
IMRT	7	35%
SIB VMAT	10	50%
3DCRT	1	5%
Conventional 2D radiotherapy	2	10%
Interval from previous RT	91.7 ± 75.1 months	
(Mean ± SD)	(Range 16–309 months)	
RT dose (mean ± SD)	43.65 ± 10.80 Gy	
	(Range 30–60 Gy)	
Reirradiated volume (mean ± SD)	255.37 ± 219.41 cc	
	(Range 17.91–781.92 cc)	
BED_3_ (mean ± SD Gy)	72 ± 17.95	

IMRT, intensity modulated radiotherapy; SIB VMAT, simultaneous integrated boost volumetric modulated arc radiotherapy; 3DCRT, 3-dimensional conformal radiotherapy; SD, standard deviation; BED_3_, biologically effective dose for *α*/*β* value 3.

**Table 3:** Toxicity profile during reirradiation.

Toxicity grades III-IV	RT dose ≤ 45 Gy	RT ≥ 46 Gy	*p* value
Radiation dermatitis	3 (15%)	1 (5%)	0.63
Mucositis	3 (15%)	1 (5%)	0.64
Xerostomia	1 (5%)	9 (45%)	0.01
Dysphagia	0 (0%)	7 (35%)	0.05
Trismus	1 (5%)	8 (40%)	0.01
